# The impact of bone cancer on the peripheral encoding of mechanical pressure stimuli

**DOI:** 10.1097/j.pain.0000000000001880

**Published:** 2020-04-13

**Authors:** Mateusz W. Kucharczyk, Kim I. Chisholm, Franziska Denk, Anthony H. Dickenson, Kirsty Bannister, Stephen B. McMahon

**Affiliations:** a Central Modulation of Pain Group, Wolfson Centre for Age-Related Diseases, King's College London, London, United Kingdom; b Neurorestoration Group, Wolfson Centre for Age-Related Diseases, King's College London, London, United Kingdom; c Department of Neuroscience, Physiology and Pharmacology, University College London, Gower Street, London, United Kingdom

**Keywords:** Cancer-induced bone pain, Silent nociceptors, Sensory coding, Pressure cuff, GCaMP6s

## Abstract

Skeletal metastases are frequently accompanied by chronic pain that is mechanoceptive in nature. Mechanistically, cancer-induced bone pain (CIBP) is mediated by peripheral sensory neurons innervating the cancerous site, the cell bodies of which are housed in the dorsal root ganglia (DRG). How these somatosensory neurons encode sensory information in CIBP remains only partly explained. Using a validated rat model, we first confirmed cortical bone destruction in CIBP but not sham-operated rats (day 14 after surgery, designated “late”-stage bone cancer). This occurred with behavioural mechanical hypersensitivity (Kruskal–Wallis H for independent samples; CIBP vs sham-operated, day 14; *P* < 0.0001). Next, hypothesising that the proportion and phenotype of primary afferents would be altered in the disease state, dorsal root ganglia in vivo imaging of genetically encoded calcium indicators and Markov Cluster Analysis were used to analyse 1748 late-stage CIBP (n = 10) and 757 sham-operated (n = 9), neurons. Distinct clusters of responses to peripheral stimuli were revealed. In CIBP rats, upon knee compression of the leg ipsilateral to the tumour, (1) 3 times as many sensory afferents responded (repeated-measures analysis of variance: *P* < 0.0001 [vs sham]); (2) there were significantly more small neurons responding (Kruskal–Wallis for independent samples (vs sham): *P* < 0.0001); and (3) approximately 13% of traced tibial cavity afferents responded (no difference observed between CIBP and sham-operated animals). We conclude that an increased sensory afferent response is present in CIBP rats, and this is likely to reflect afferent recruitment from outside of the bone rather than increased intraosseous afferent activity.

## Abstract 2

Supplemental Digital Content is Available in the Text.

In vivo GCaMP6s imaging of hind-limb sensory neurons revealed that the number of mechanically responsive sensory neurons triples in bone cancer conditions.

## 1. Introduction

Severe pain accompanies many different cancer types^5,19^ and is particularly prominent in cases where malignant tumours have invaded the skeleton.^18,25^ In the clinic, cancer-induced bone pain (CIBP) often manifests as a result of musculoskeletal compression, both due to weight bearing in moving subjects and increased intraosseous pressure secondary to tumour expansion.^4,6–8,17,20–24,26,35^ The pain is therefore difficult to manage in mobile patients, who suffer a high incidence of breakthrough pain.^4,11,17^ Current analgesic options offer limited respite for such sufferers.^11,17,19^

Mechanistically, transmission of sensory information from the bone to central sites in CIBP is through a genetically and physiologically heterogenous population of primary afferent sensory neurons.^3,16,28,31,36,37,39^ The mechanism(s) by which these pseudounipolar somatosensory neurons encode stimulus intensity on the population level remains a topic of investigation.^28,37^ Two different types of mechanism are hypothesised: frequency coding (a number of action potentials fired in response to a given stimulus strength) and population coding (the number of afferents engaged in response to a given stimulus). Using in vivo imaging of genetically encoded calcium indicators, which offers the ability to sample large neuronal populations, others have reported that heating and cooling sensations are encoded differently by primary afferents.^37^ Pressure and mechanosensation coding has been investigated on both a molecular^29,32^ and neuronal level.^28,37^ Despite pioneering work, many questions remain regarding afferent function both in health and disease. How is modality-reflecting information encoded by sensory neurons? And are their functional responses altered in disease conditions?

To investigate such unknowns, we generated a validated rat model of CIBP using syngeneic mammary gland carcinoma cells.^20^ Tumour progression was monitored using microcomputer tomography reconstruction of the rat tibiae at 2 different time points: days 7/8 and days 14/15. Trabecular bone damage, evident at both time points, and cortical bone impairment, evident only at days 14/15, were suggestive of early- vs late-stage modelling of CIBP in the model, and bone damage was related to animal behaviour. Subsequently, using in vivo functional imaging of primary somatosensory afferent neurons in late-stage CIBP or sham-operated rats, we sought to investigate how mechanical stimuli are encoded during compression or altered limb position and whether these modalities are encoded by musculoskeletal afferents. Crucially, by comparing functional responses in healthy control and late-stage CIBP rats using an unsupervised clustering of neuronal responses, we could reveal major responder classes to defined stimuli, allowing for investigation whether or not primary afferent functional responses are altered in disease conditions.

## 2. Materials and methods

### 2.1. Cells

Syngeneic rat mammary gland adenocarcinoma cells (MRMT-1; Riken cell bank, Tsukuba, Japan), isolated from female Sprague–Dawley rat, were cultured in RPMI-1640 medium (Invitrogen, Paisley, United Kingdom) supplemented with 10% foetal bovine serum, 1% L-glutamine, and 2% penicillin/streptomycin (Invitrogen). All cells were incubated at 5% CO_2_ in a humidity-controlled environment (37°C; Forma Scientific, Loughborough, United Kingdom). On the day of surgery, MRMT-1 cells were released by brief exposure to 0.1% wt/vol trypsin-ethylenediaminetetraacetic acid and collected by centrifugation in medium for 5 minutes at 1000 rpm. The pellet was washed with Hanks' balanced salt solution (HBSS) without calcium, magnesium, or phenol red (Invitrogen) and centrifuged for 5 minutes at 1000 rpm. MRMT1 cells were suspended in HBSS to a final concentration of 300,000 cells/mL and kept on ice until use. Only live cells were counted with the aid of trypan blue (Sigma, Gillingham, United Kingdom) staining. Cell viability after incubation on ice was checked after surgery, and no more than 5% to 10% of cells were found dead after 4 hours of ice storage.

### 2.2. Animals

Male Sprague–Dawley rats (UCL Biological Services, London, United Kingdom or Charles-River, United Kingdom) were group housed on a 12:12-hour light–dark cycle. Food and water were available ad libitum. Animal house conditions were strictly controlled, maintaining stable levels of humidity (40%-50%) and temperature (22 ± 2°C). All procedures described were approved by the UK Home Office and conformed to the Animals (Scientific Procedures) Act 1986 and ARRIVE guidelines.^15^ Every effort was made to reduce animal suffering, and the number of animals used was in accordance with International Association for the Study of Pain (IASP) ethical guidelines.^40^

### 2.3. Administration of tracers and calcium indicators

Rats weighing 60 to 70 g were anaesthetised using isoflurane (1.5%-2% in oxygen, Piramal, Morpeth, United Kingdom) and maintained at around 37°C using a homeothermic heating mat and 50 μL of meloxicam (2 mg/kg, Metacam; Boehringer Ingelheim, Berkshire, United Kingdom) was subcutaneously administered for postoperative pain management. Animals were fixed in a stereotaxic apparatus (David Kopf Instruments, Tujunga, CA), their lumbar region was clamped, and spinal T12-L1 intervertebral space was exposed by bending the lumbar region rostrally providing easy access to the underlaying dura without the need for laminectomy. A small puncture in the dura was made, and a thin catheter of 0.2-mm diameter (Braintree Scientific, Braintree, MA) was inserted in the caudal direction. Ten microliter of AAV9.CAG.GCaMP6s.WPRE.SV40 was infused into the spinal T12-L1 intrathecal space (titer ≥ 1 × 10^13^ vg/mL, a gift from Douglas Kim & GENIE Project via Addgene viral prep #100844-AAV9, United States^2^). The catheter was left in place for 2 minutes before slow withdrawal. The incision was closed with wound clamps and postsurgical glue (Vetabond, 3M; Bracknell, United Kingdom). After 7 days, the left tibia was injected with 5 μL of 4% Fast Blue neuronal tracer (Polysciences, Inc, Hirschberg an der Bergstraβe, Germany) in saline, and the muscle layer adjacent to the Fast Blue injected bone was injected with 5 μL of pAAV-CAG-tdTomato (titer 7 × 10^12^ vg/mL, a gift from Edward Boyden via Addgene viral prep #59462-AAVrg, United States). After a 7-day recovery period, animals were randomly divided into 2 groups. After a 7-day recovery period, animals were randomly divided into 2 groups receiving either cancer cells or sham HBSS buffer treatment into the left tibia (through the same hole in the bone to prevent further damage).

### 2.4. Cancer cell or sham surgery

A validated rat CIBP model was generated as described previously.^20^ Briefly, rats weighing 120 to 140 g (for late-stage CIBP, 14 days after surgery) or 180 to 200 g (for early-stage CIBP, 7 days after surgery) were anaesthetised using isoflurane (induction 5%, maintenance 1.5%-2% in 2 L/min O_2_) and, after subcutaneous perioperative meloxicam injection (50 μL 2 mg/kg), were subjected to the surgical procedure of cancer cell implantation into the right tibiae. In aseptic conditions, a small incision was made on a shaved and disinfected area of the tibia's anterior-medial surface. The tibia was carefully exposed with minimal damage to the surrounding tissue. Using a 0.7-mm dental drill, a hole was made in the bone through which a thin polyethylene tube (I.D. 0.28 mm, O.D. 0.61 mm; Intramedic, Becton Dickinson and Co, Sparks, MD) was inserted 1 to 1.5 cm into the intramedullary cavity. Using a Hamilton syringe, either 3 × 10^3^ MRMT-1 carcinoma cells in 10-μL HBSS or 10-μL HBSS alone (sham) were injected into the cavity. The tubing was removed, and the hole plugged with bone restorative material (IRM, Dentsply, Surrey, United Kingdom). The wound was irrigated with saline and closed with Vicryl 4-0 absorbable sutures and wound glue (VetaBond 3M, United Kingdom). The animals were placed in a thermoregulated recovery box until fully awake. After 2 weeks, animals were subjected to terminal in vivo calcium imaging (250-280 g on the day of imaging).

### 2.5. Static weight bearing

Weight-bearing behaviour was assessed before surgery (day 0) and at 2, 7, and 14 days after surgery. The force exerted by each hind paw was measured 5 times with a 10- to 20-second gap between measurements. Measurements from each paw were averaged, and the percentage of weight born on each side was calculated.

### 2.6. In vivo calcium imaging of sensory neurons

Rats were anaesthetised (using urethane, 12.5% wt/vol in saline, Sigma), and core body temperature was maintained with a homeothermic heating mat. The muscle overlying the L3, L4, and L5 vertebral segments and the bone around either the L3 or L4 DRG was removed to expose the underlying epineurium and dura mater over the DRG. The position of the animal's body was varied between prone and lateral recumbent to orient the DRG in a more horizontal plane. The exposure was then stabilised at the neighbouring vertebrae using spinal clamps (Precision Systems and Instrumentation, Fairfax Station, VA) attached to a custom-made imaging stage. The exposed cord and DRG were covered with silicone elastomer (World Precision Instruments, Ltd, Hertfordshire, United Kingdom) to avoid drying and to maintain a physiological environment. The rat was then placed under the Eclipse Ni-E FN upright confocal/multiphoton microscope (Nikon, Surbiton, United Kingdom), and the microscope stage was variably diagonally orientated to optimise focus on the DRG. The ambient temperature during imaging was kept at 32°C throughout. All images were acquired using a 10× dry objective. To obtain confocal images, a 488-nm argon ion laser line was used. GCaMP signal was collected at 500 to 550 nm. Time series recordings were taken with an in-plane resolution of 512 × 512 pixels and a fully open pinhole for video-rate acquisition. Image acquisition varied between 2 to 4 Hz depending on the experimental requirements and signal strength. At the end of the experiment, rats were sacrificed by clamping the trachea tube, left for 1 hour for the DRG to fill up with calcium for maximum signal control.

### 2.7. Activation of sensory neurons for GCaMP in vivo imaging

Mechanical stimulation consisted of (1) innocuous brushing of the leg, (2) leg stretching, and (3) pressure application (through small pressure cuffs, Neonate Disposable BP Cuff, Size #2, E-Medical Medical Supplies, Rhymney, United Kingdom) connected to a manometer and air pump and positioned over the knee-tibial head, or calf, or calf-ankle, to deliver incremental pressure (50-mm Hg increments every 10 seconds, in the range of 0-400 mm Hg).

### 2.8. Calcium imaging data analysis and Markov cluster analysis

Drift in time-lapse recordings was corrected using NIS Elements AR 4.30.01 (Nikon, align application). Image processing was performed using Fiji/ImageJ Version 1.52 hours and written in house scripts in R (RStudio, Version 1.1.419). Further analysis, graphing, and statistical evaluation were undertaken with a combination of Microsoft Office Excel 2013, IBM SPSS Statistics 25 package and RStudio. To generate traces of calcium signals from time-lapse images, regions of interest (ROIs) surrounding cell bodies were chosen using a free hand selection tool in Fiji. Regions of interest were chosen with minimal overlap to ensure less interference from surrounding somata. A region of background was selected, and its signal subtracted from each ROI. To generate normalised data, a baseline period of fluorescence was recorded for each ROI, and changes from this baseline fluorescence were calculated as ΔF/F and expressed in percentages.^3^ Implemented here are stringent criteria, where an average signal reaching 70% above baseline fluorescence plus 4 SDs was qualified as a response. Percentage of responders was quantified in a binary fashion within all selected ROIs. The fluorescence intensity and size analysis were performed only for responders. Nonresponding cells were not analysed for their fluorescence intensity levels because it would artificially introduce biased zero values for these cells, which were either nonresponding for the particular modality or were outside of the stimulated receptive field. Thus, only those cells were analysed for intensity and size, which responded at least once to the given stimulus modality (ie, knee compression 0-400 mm Hg).

### 2.9. Markov Cluster Analysis

Markov Cluster Analysis was used to cluster hundreds of neurons responding to the predefined stimuli.^10^ BioLayout Express (under GNU Public License, Kajeka Ltd, Edinburgh, United Kingdom) was used to run the analysis.^33^ The data derived from the range of neuronal responses (dF/F values averaged for each stimulus length, ie, all frames from the entire duration of the 50 mm Hg compression) to the defined stimuli, originating from all responders across the time. Constructed csv files with all responders' values were used to construct network graphs (see step 1 in Fig. S1A, available at http://links.lww.com/PAIN/A994). Initially, the similarity between individual cell responses was determined by the Pearson correlation. Pairwise Pearson correlation coefficients were calculated for every cell set after defined sensory stimuli, and correlation coefficients above a predefined threshold (*R* > 0.9) were used to draw edges between cells (nodes) in the construction of network graphs. The nodes over the preselected value were removed from the graph (see step 2 in Fig. S1A, available at http://links.lww.com/PAIN/A994). Several trials with different *R*-values were tested, and the value of 0.9 was selected as the best “trade-off” between overpopulated graphs with overwhelming number of clusters and no biological meanings (*R* < 0.85) and exclusion or too many cells from the analysis (*R* > 0.9). Next, the generated nonweighted graphs were clustered with the MCA with the following parameters: preinflation = 1.8, inflation = 1.8, scheme = 3, and minimal number of clusters = 5. The most restrictive parameter—inflation (defines granularity of the clustering) was chosen experimentally to most tightly represent clean clusters without losing too many cells from the analysis (see step 3 in Fig. S1A, available at http://links.lww.com/PAIN/A994). Post hoc analysis of all visualised clusters allowed for user-defined merging of clusters with biologically relevant similarity. This last part was supervised (see step 4 in Fig. S1A, available at http://links.lww.com/PAIN/A994). Final core data were exported with cluster's tag for each cell.

### 2.10. Principal Component Analysis

A csv file containing final core data after MCA was used in principal component analysis to demonstrate that the classical analysis of variances (ANOVAs) is unable to detect different patterns in the longitudinal data sets (ie, fluorescence changes to the pressure ramp across hundreds of cells). Principal component analysis was run in R (RStudio, Version 1.1.419), and analysis and visualisation were performed using the following packages: factoextra, corrplot, FactoMineR, and ggfortify (Figs. S1B, C, D, available at http://links.lww.com/PAIN/A994).

### 2.11. Immunohistochemistry

At the end of each GCaMP imaging experiment, L1-L5 ipsi/contra DRG were collected, postfixed in 4% paraformaldehyde, cryosectioned to 10 μm thick slices, and incubated overnight with primary antibodies against green fluorescent protein (chicken, 1:1000, ab13970; Abcam, Cambridge, United Kingdom), Calcitonin gene-related peptide (CGRP) (mouse, 1:1000, ab81887; Abcam), and IB4 (conjugated to Alexa Fluor 647; 1:250, I32450, Thermo Fisher Scientific, Loughborough, United Kingdom). Slides were then incubated with the appropriate fluorophore-conjugated secondary antibodies (all from Invitrogen, Eugene, OR). Samples were imaged with an LSM 710 laser-scanning confocal microscope (Carl Zeiss Microscopy GmbH, Oberkochen, Germany) using 10× (0.3 NA) and 20 × (0.8 NA) dry objectives and analysed with Fiji Win 64.

### 2.12. Microcomputed tomography of cancer-bearing legs

Rat tibiae, cleared of excess muscle and soft tissue, were placed into a microcomputed tomography (CT) scanner (Skyscan1172, Bruker, Coventry, United Kingdom) with Hamamatsu 10 Mp camera. Recording parameters were set as follows: source voltage at 40 kV, source current at 250 μA, rotation step at 0.600°, with 2 frames averaging, and 0.5-mm aluminium filter. For reconstruction, NRecon software (version: 1.6.10.4) was used. In total, over 500, 34-μm-thick virtual slices were collected per bone. Because the reference point for rat's tibia for micro-CT analysis was not previously described, we established an anatomically relevant reference point, which was not affected by cancer, from which a region of interest was chosen for further analysis. Reference point for bone mineral density (BMD) analysis was defined as the internal tip of the intercondylar area, which was consistently located at 5 mm from the centre of the cancer growth zone. For BMD, the cancer growth zone encompassing space between 3 and 7 mm caudally from the reference point was quantified. A total of 119 scanned planes, each with a thickness of 34 μm, were analysed (see Fig. S2A for more details, available at http://links.lww.com/PAIN/A994). Comparison with 2 known density standards allowed us to quantify BMD values in mg·cm^−3^ of both trabecular and cortical bone (using dataViewer software). Other bone topography parameters (Fig. S2B, available at http://links.lww.com/PAIN/A994) were calculated for exactly the same 119 scanned planes as for BMD using BoneJ plugin from Fiji.^9^ Representative visualisations were prepared with Fiji with 3D viewer plugin.

### 2.13. Quantification and statistical analysis

Statistical analyses were performed using SPSS v25 (IBM, Armonk, NY). All data plotted represent mean ± SEM. Detailed description of the number of samples analysed, and their meaning, together with values obtained from statistical tests, can be found in each figure legend. Main effects from ANOVAs are expressed as an F-statistic and *P*-value within brackets. Statistical differences in the neuronal responses from the GCaMP experiment were determined using a 2-way repeated-measures ANOVA (RM-ANOVA), where applicable with Bonferroni post hoc test. Kruskal–Wallis one-way ANOVA test (K-W, one-way ANOVA) was used to analyse behavioural data for weight bearing and body mass between sham and CIBP groups. Unpaired *t* test was used to compare number of responders in each cluster between sham and CIBP groups. One-way ANOVA with Tukey post hoc performed in GraphPad Prism was used to analyse data for BMD.

## 3. Results

### 3.1. Cancer progression impacts bone integrity and correlates with mechanical hypersensitivity

**Figure 1.**
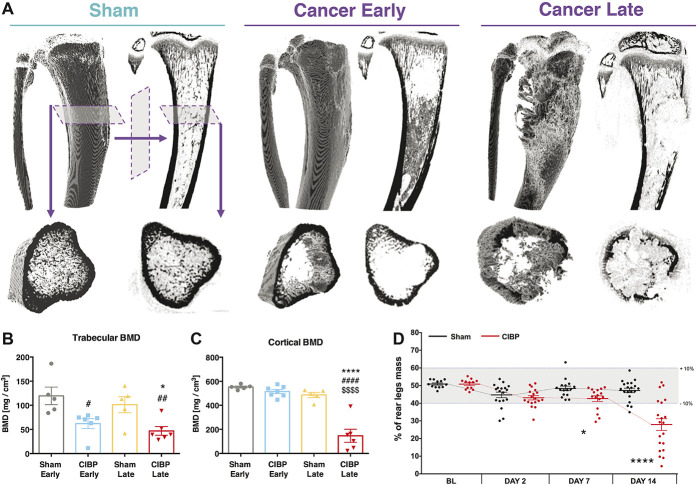
The impact of cancer progression on bone innervation. Example microcomputer tomography reconstructions of rat tibiae. Panels depict a sham-operated control, an early (day 7/8) and a late (day 14/15) cancer stage. Top panels represent a whole 3D-rendered tibia with corresponding orthogonal projection. Bottom panel shows a plantar representation of selected microscans from the top panel. An early cancer stage is characterised by the trabecular bone lesions, whereas the late stage by both trabecular and cortical bone lesions (A). Trabecular bone mineral density quantification is shown (volumetric bone mineral density quantification from 114 reconstructed microscans (every 34 µm) per bone. Selected planes for analysis were chosen to cover the tumour growth area (see methods for details). Each dot represents a single bone from a separate animal. Data represent the mean ± SEM (in mg/cm^3^). One-way ANOVA with Tukey post hoc test: *or #*P* < 0.05, ##*P* < 0.01. #vs early sham, *vs respective sham (B). Cortical bone mineral density quantification is shown. Data represent the mean ± SEM (in mg/cm^3^). One-way ANOVA with Tukey post hoc test: ****P < 0.0001. #vs early sham, *vs late sham, $vs early cancer (C). Static weight-bearing measurement of rear legs. Within a timepoint, each dot represents a single animal (n = 13-20 per group). Each measurement was taken as an average of 5 consecutive readouts per animal per timepoint. All data represent the mean ± SEM. Kruskal–Wallis H for independent samples: *P < 0.05, ****P < 0.0001 vs respective sham (D). See also Figs. S2 and S3, available at http://links.lww.com/PAIN/A994. ANOVA, analysis of variance.

Bone damage was assessed using a microcomputer tomography technique at days 7/8 and 14/15 after surgery (Fig. 1A). Significant damage to the trabecular bone was observed in both sham-operated and CIBP early- and late-stage rat groups (one-way ANOVA [group]: F_3, 18_ = 6.272, *P* = 0.0042, Tukey post hoc: CIBP early vs sham early *P* < 0.01, CIBP late vs sham late or vs sham early *P* < 0.05) (Fig. 1B). Only cortical bone integrity was compromised at the later time stage (485 mg/cm^3^ in the sham-operated rat group compared with 146 mg/cm^3^ in the CIBP late-stage group) (one-way ANOVA [group]: F_3, 19_ = 35.57, *P* < 0.0001, Tukey post hoc: CIBP late vs all the other groups *P* < 0.0001) (Fig. 1C). Volumetric reconstruction enabled BMD quantification for the whole tumour growth area (Fig. S2A, available at http://links.lww.com/PAIN/A994). Other topographical bone parameters were affected also (Fig. S2B, available at http://links.lww.com/PAIN/A994). Although body weight gain remained stable in all groups (Kruskal–Wallis H for independent samples [cancer vs sham]: day 0: χ^2^(1) = 0.188, *P* = 0.665, day 2: χ^2^(1) = 1.503, *P* = 0.220, day 7: χ^2^(1) = 2.945, *P* = 0.086, day 14: χ^2^(1) = 1.087, *P* = 0.297) (Fig. S3A, available at http://links.lww.com/PAIN/A994), the behavioural data demonstrate that CIBP rats manifest mechanical hypersensitivity; significant changes in static weight bearing between rear legs were evident from day 7 after surgery (Kruskal–Wallis H for independent samples [cancer vs sham]: day 0: χ^2^(1) = 0.083, *P* = 0.773, day 2: χ^2^(1) = 1.602, *P* = 0.206, day 7: χ^2^(1) = 5.286, *P* = 0.022, day 14: χ^2^(1) = 15.384, *P* < 0.0001) (Fig. 1D).

### 3.2. The number of mechanically responsive sensory neurons triples in cancer-induced bone pain rats

**Figure 2.**
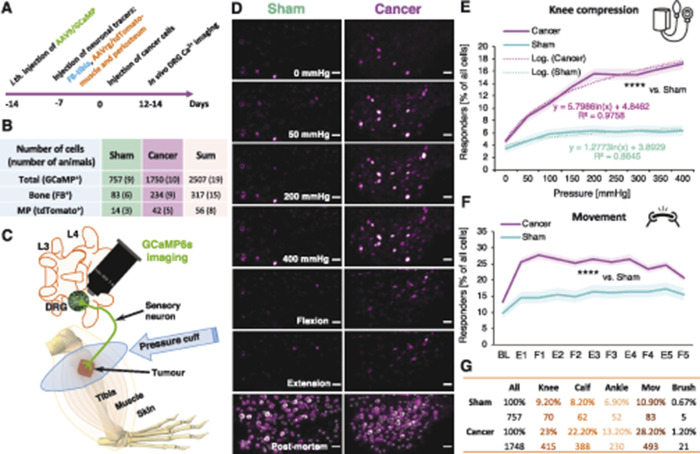
The number of mechanically responsive sensory neurons is tripled in animals with bone cancer. The experimental timeline is shown. The injection of neuronal tracers and cancer cells implantation was performed through the same hole in the tibia to limit damage (A). A summary table representing the number of cells analysed in the in vivo calcium imaging experiment, in brackets are the numbers of animals studied (B). Schematic representation of the in vivo GCaMP6s imaging (C). Selected video frames of imaged DRG cell bodies responding to the knee compression and leg movement (flexion/extension along the body axis). Representative frames from the end of an in vivo imaging session with circled DRG neuronal cell bodies taken for analysis as a denominator of all imaged DRG neurons in the selected field of view. The confocal z-scan was taken over 30-minute post mortem allowing for calcium to build up in the neuronal cell bodies permitting indication of most of the GCaMP6s-labelled cells. Sham-operated (left) and CIBP (right). Scale bars, 100 µm (D). Percentage of responders from 2505 neuronal cell bodies analysed from L3 and L4 DRG during knee compression in sham and CIBP animals. Data represent the mean ± SEM (shaded areas) of n = 757 cells (sham) from 9 animals, and n = 1748 cells from 10 animals (CIBP). Repeated-measures ANOVA with Bonferroni correction: ****P < 0.0001 (vs sham) (E). Percentage of responders from 1592 neuronal cell bodies analysed from L3 and L4 DRG during the gentle leg movement along the body axis in sham and CIBP animals. Responses of 5 consecutive extension (E) and flexion (F) pairs are presented. Data represent the mean ± SEM (shaded areas) of n = 359 cells (sham) from 6 animals, and n = 1233 cells from 7 animals (CIBP). Repeated-measures ANOVA with Bonferroni correction: ****P < 0.0001 (vs sham) (F). Table representing numbers of responders after each stimulus in both analysed groups. Note that the compression of the tumour-bearing area (“knee”) results in the highest number of responding cells (G). See also Movie 1 (available at http://links.lww.com/PAIN/A993). FB, Fast Blue; GCaMP6s, genetically encoded calcium indicator; AAV9, adeno-associated virus serotype 9; AAVrg, adeno-associated virus serotype 2 retrograde; ANOVA, analysis of variance; CIBP, cancer-induced bone pain; DRG, dorsal root ganglia, L3, L4, lumbar vertebrae 3 and 4, respectively.

Because cortical BMD was reduced only in late-stage CIBP rats, all data considered forthwith are from these and equivalent (late-stage) sham-operated rats. An experimental timeline for injection of neuronal tracers and cancer cell implantation is provided (Fig. 2A). In total, 757 DRG neuronal cell bodies from 9 sham-operated rats, and 1748 neurons from 10 CIBP animals, were analysed (Fig. 2B). A subset of video frames of imaged DRG cell bodies responding to the knee compression and the leg movements (flexion/extension) and a schematic representation of the in vivo GCaMP6s imaging are shown (Figs. 2C and D).

Lumbar DRG L3 and L4 were chosen for imaging based on Fast Blue (FB) tracing (Figs. S3B, C, available at http://links.lww.com/PAIN/A994; and^14^), and 6 to 7 L3 DRG and 3 L4 DRG from each group were imaged and lumbar level responses pooled. The percentage of responding cells in sham-operated vs CIBP rats after knee (tumour-growth area) compression indicated a 3-fold increase in the number of responders in CIBP rats (around 18%) compared with their sham-operated counterparts (around 6%) (RM-ANOVA: F_1, 2503_ = 43.276, *P* < 0.0001 [vs sham]). Interestingly, increased compression force was reflected in the recruitment of responders in the CIBP group in an approximately logarithmic fashion (Fig. 2E). There seemed to be a threshold between 50 to 100 mm Hg, after which all potential mechanoceptors within the imaged field of view were responding to the chosen receptive field stimulation. CIBP rats also demonstrated a 2-fold increase in primary afferent responders after limb movement (without weight bearing) compared with sham-operated rats (n = 359 cells [sham] from 6 animals, and n = 1233 cells from 7 animals [cancer]; RM-ANOVA: F_1, 1590_ = 17.396, *P* < 0.0001 [vs sham]) (Fig. 2F). Differences were also seen following pressure cuff application to receptive fields located further away from the cancer growth area (ie, calf and ankle—these were all statistically significant: RM-ANOVA [calf]: F_1, 2242_ = 4.664, *P* < 0.05 [vs sham] and RM-ANOVA [ankle]: F_1, 2188_ = 6.967, *P* < 0.01 [vs sham]). By contrast, no significant difference in the number of primary afferent responders to dynamic brushing of the ipsilateral hind limb was observed between sham-operated and CIBP animals (Student *t*-test: *P* = 0.376) (Fig. 2G).

### 3.3. Pressure is encoded by small to medium size neurons

**Figure 3.**
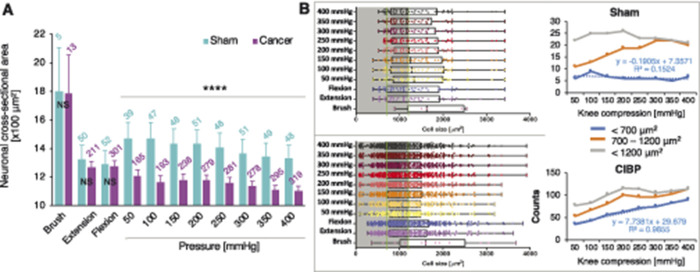
More small-diameter afferents respond to increasing leg compression. Cell size analysis of all responders during dynamic brushing of the leg surface, leg movement along the body axis (first pull and push shown), and knee compression in sham and cancer animals. Pressure was increased in 50-mm Hg steps every 10 seconds. Data represent the mean ± SEM (shaded areas). Number of cells analysed are above each bar. Brush, extension, and flexion (first pair): unpaired *t* test: NS, nonsignificant, knee compression: Kruskal–Wallis for independent samples: ****P < 0.0001 (vs sham) (A). Left panels depict cell size distributions in sham (top) and CIBP (bottom) of all responders to different stimuli. Green lines highlight cell size separators used: <700 µm^2^ (small cells), >700 < 1200 µm^2^ (medium cells). Right panels show a summary count of responders to the increased knee compression within each cell size range. Sham (top), CIBP (bottom). Note that the number of small-diameter responders increase linearly with the pressure surge (B). CIBP, cancer-induced bone pain.

We analysed responder cell size distribution in sham-operated and CIBP rats (choosing 700 and 1200 µm^2^ to define the upper and lower limits of medium-sized cells). In sham-operated rats, the number of medium-sized responders increased with increasing stimulus pressure (knee compression: Kruskal–Wallis for independent samples: F_1, 2429_ = 28.469, *P* < 0.0001) suggesting that pressure is encoded mainly by small- to medium-sized neurons, while dynamic brushing only recruited few large-sized neurons (brush: unpaired *t* test: *P* = 0.984) (Figs. 3A and B). A decrease in average responding cell size with increased compression force was observed (Figs. 3A and B), and there were significant differences in the average cell sizes of responders to knee compression (Kruskal–Wallis for independent samples [sham vs cancer]: F_1, 2429_ = 28.469, *P* < 0.0001) (Fig. 3A), but not brush (unpaired *t* test: *P* = 0.984) or leg movement (Kruskal–Wallis for independent samples [sham vs cancer]: F_1, 674_ = 0.110, *P* = 0.740) (Fig. 3A), between CIBP and sham animals. In CIBP rats, an additional population of small diameter neurons (likely C nociceptors) was activated proportionally with increasing stimulus strength, reaching almost 3 times the number of cells that responded to the 400 mm Hg than the initial 50 mm Hg (Fig. 3B).

### 3.4. Leg compression and position are differentially coded by dorsal root ganglion sensory neurons

**Figure 4.**
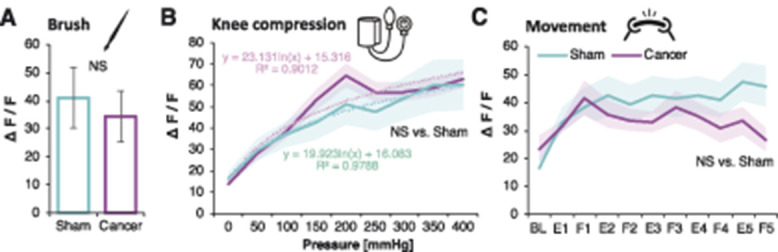
Responders GCaMP fluorescence intensity is not affected by bone cancer. Normalised fluorescence intensities of all responding neuronal cell bodies from L3 and L4 DRG to dynamic brushing of the leg. Data represent the mean ± SEM. n = 5 cells (sham) from 6 animals, and n = 21 cells from 6 animals (CIBP). Unpaired *t* test: NS, nonsignificant (A). Normalised fluorescence intensities of all responding neuronal cell bodies from L3 and L4 DRG to knee compression. Pressure was increased in 50-mm Hg increments every 10 seconds. Data represent the mean ± SEM (shaded areas). n = 70 cells (sham) from 9 animals, and n = 415 cells from 10 animals (CIBP). Repeated-measures ANOVA: NS (vs sham) (B). Normalised fluorescence intensities of all responding neuronal cell bodies from L3 and L4 DRG to leg movement along the body axis (F, flexion; E, extension). Data represent the mean ± SEM (shaded areas), n = 83 cells (sham) from 6 animals, and n = 493 cells from 7 animals (CIBP). Repeated-measures ANOVA: NS (vs sham) (C). ANOVA, analysis of variance; CIBP, cancer-induced bone pain; DRG, dorsal root ganglia.

**Figure 5.**
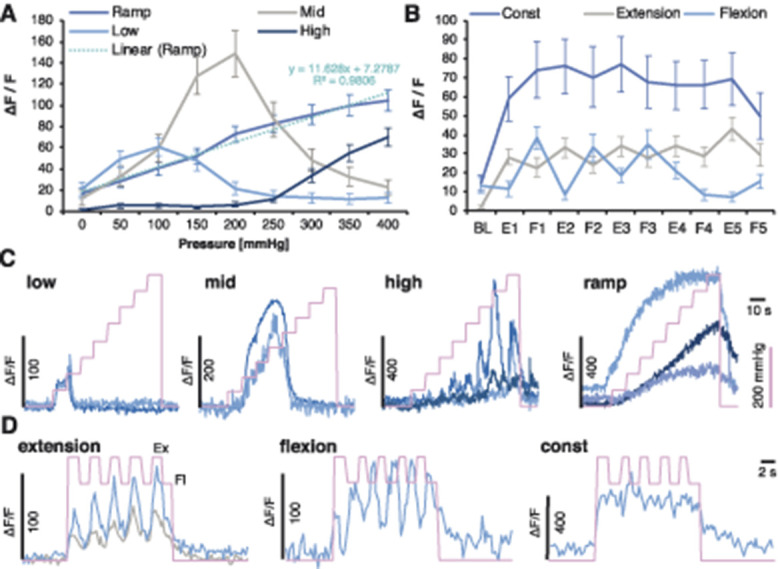
Leg compression and position are differentially coded by DRG sensory neurons. Unsupervised clustering of neuronal responses to the knee compression. Markov Cluster Analysis (MCA) revealed 4 major clusters of responders to knee compression depicted as “Ramp,” “Low,” “Mid,” and “High.” The “Ramp” cluster could be fitted by linear regression (A). MCA revealed 3 major clusters of cells depicted as “Const,” “Flexion,” or “Extension” following leg movement. Because of the lack of differences in the clusters between the sham and cancer animals, groups were pooled (Fig. 6) (B). Example normalised GCaMP6s fluorescence traces from DRG neurons of the identified knee compression clusters. Pink line indicates the surge in cuff pressure (C). Example normalised GCaMP6s fluorescence traces from DRG neurons of the identified movement-evoked clusters. Pink line indicates events: higher points are reflecting extension (Ex) and lower flexion (Fl), the leg along the body axis (D). DRG, dorsal root ganglia.

**Figure 6.**
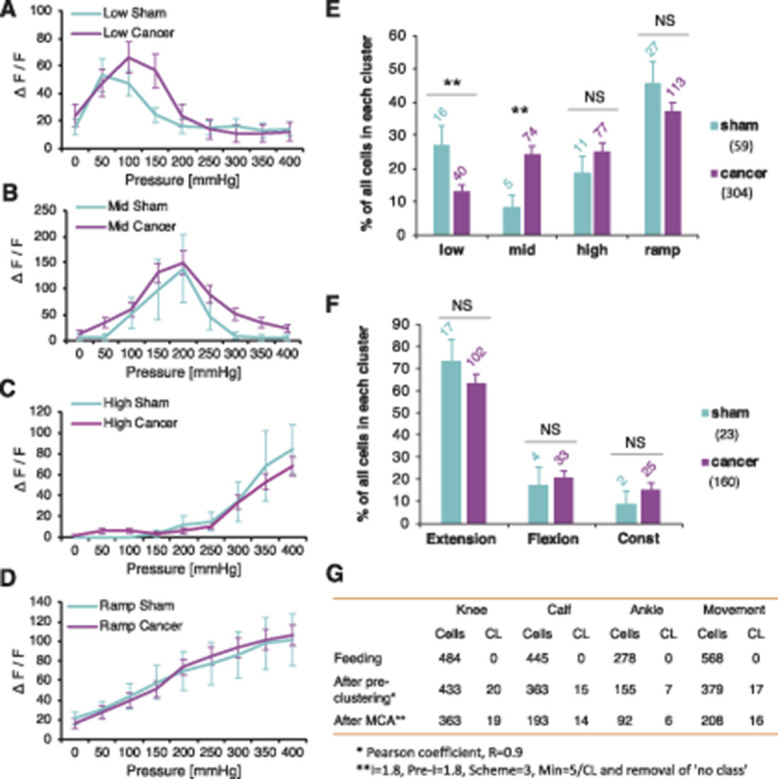
Leg compression and position are differentially coded by DRG sensory neurons. “Low” cluster of neuronal responses to the knee compression revealed by Markov Cluster Analysis (MCA) with respect to the treatment group. n = 16 cells (sham) from 3 animals, and n = 40 cells from 10 animals (cancer). Repeated-measures ANOVA: nonsignificant (vs sham) (A). “Mid” cluster of neuronal responses to the knee compression revealed by Markov Cluster Analysis with respect to the treatment group. n = 5 cells (sham) from 4 animals, and n = 74 cells from 10 animals (cancer). Repeated-measures ANOVA: nonsignificant (vs sham) (B). “High” cluster of neuronal responses to the knee compression revealed by Markov Cluster Analysis with respect to the treatment group. n = 11 cells (sham) from 2 animals, and n = 77 cells from 10 animals (cancer). Repeated-measures ANOVA: nonsignificant (vs sham) (C). “Ramp” cluster of neuronal responses to the knee compression revealed by Markov Cluster Analysis with respect to the treatment group. n = 27 cells (sham) from 3 animals, and n = 113 cells from 10 animals (cancer). Repeated-measures ANOVA: nonsignificant (vs sham) (D). Percentage of cells in each knee compression cluster with regards to the group classifiers is shown. Data represent the mean ± SEM. Numbers over the bars represent total numbers of cells identified in each cluster. Total numbers of cells after MCA in sham and cancer groups are given in brackets. Unpaired *t* test: **P < 0.01, NS, nonsignificant (E). Percentage of cells in each movement cluster with regards to the group classifiers is shown. Data represent the mean ± SEM. Numbers over the bars represent total numbers of cells identified in each cluster. Total numbers of cells after MCA in sham and cancer groups are given in brackets. Unpaired *t* test: NS, nonsignificant (F). Summary table of all responders after each step of MCA performed with respect to different stimulus (See Fig. S2A, available at http://links.lww.com/PAIN/A994). CL reflects number of clusters present at each step of the analysis before final manual merging (G). Data represent the mean ± SEM. ANOVA, analysis of variance; DRG, dorsal root ganglia.

Contrary to cell number, the fluorescence intensity of responding cells was not altered between sham and cancer groups after brushing (unpaired *t* test vs sham: *P* = 0.737), knee compression (RM-ANOVA [vs sham]: F_1, 483_ = 0.177, *P* = 0.674), or leg movement (RM-ANOVA [vs sham]: F_1, 574_ = 0.306, *P* = 0.580) (Figs. 4A–C). However, fluorescence intensity increased proportionally with pressure intensity in both sham-operated and CIBP rats (Figs. 4A–C). The major responder pattern of firing was analysed using Markov Cluster Analysis (MCA) (Fig. S1A, available at http://links.lww.com/PAIN/A994). This largely unsupervised approach revealed 4 main clusters of neuronal responses to limb compression where afferent responses were classified as (1) triggered by “low” pressure (<100 mm Hg), (2) triggered by “middle” pressure (peak at around 200 mm Hg), (3) triggered by “high” pressure (>300 mm Hg), or (3) triggered by the pressure surge (0-400 mm Hg, “ramp”) (Figs. 5A and C). Neuronal responses to limb movement revealed 3 clusters, classified as a fluorescence increase to limb (1) extension, (2) flexion, or (3) movement (“const”) (Figs. 5B and D). Again, no differences were seen between sham and CIBP fluorescence intensity in each cluster (Figs. 6A–D, movement not shown). However, there were significantly more cells in the mid, and less in the low, clusters in CIBP rats (unpaired *t* test: “Low” *P* = 0.007, “Mid” *P* = 0.007, “High” *P* = 0.274, and “Ramp” *P* = 0.216) (Fig. 6E). Conversely, there was no difference in the number of responders in the movement clusters (unpaired *t* test: Extension *P* = 0.342, Flexion *P* = 0.720, and “Const” *P* = 0.384) (Fig. 6F). The number of responders after each MCA step is displayed for clarity (Fig. 6G).

### 3.5. Tibial afferent function in health and bone cancer

**Figure 7.**
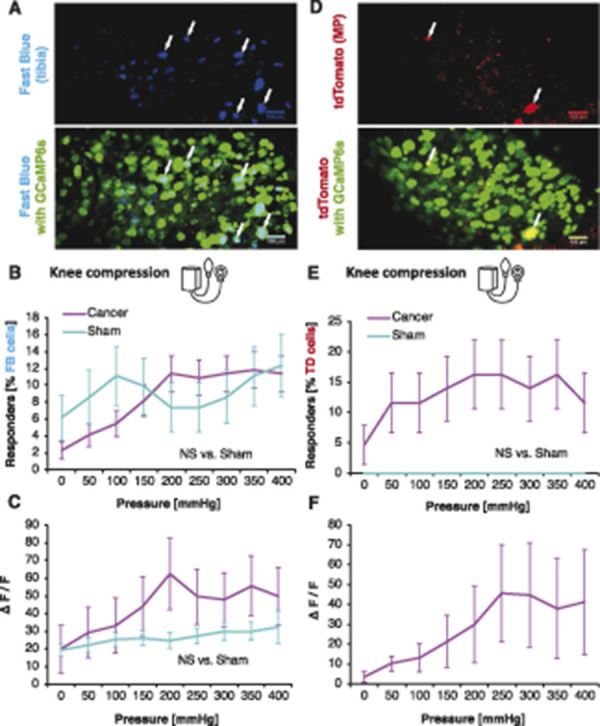
Intratibial and peritibial afferent function in health and bone cancer. Fast Blue (FB)-traced neurons from the tibial cavity. Representative z-stack collected at the end of the in vivo imaging experiment to identify traced cells (blue) and GCaMP6s (green). Scale bars: 100 µm. Arrows indicate FB-traced neurons (A). Percentage of responders of all FB-traced tibial afferents within L3 and L4 DRG during knee compression in sham and CIBP rats. Data represent the mean ± SEM of n = 81 cells from 6 animals (sham) and 220 cells from 9 animals (cancer). Repeated-measures ANOVA: NS, nonsignificant (B). Fluorescence intensities of all responding FB+ neuronal cell bodies to knee compression. Pressure was increased in 50-mm Hg increments every 10 seconds. Data represent the mean ± SEM of n = 15 cells (sham) from 6 animals, and n = 34 cells from 9 animals (CIBP). Repeated-measures ANOVA: NS, nonsignificant (C). AAVrg/tdTomato (TD)-traced neurons innervating the muscle and periosteum (MP) surrounding tibia. Representative z-stack collected at the end of the in vivo imaging experiment to identify traced cells (red) and GCaMP6s (green). Scale bars: 100 µm. Arrows indicate TD-traced neurons (D). Percentage of responders of all TD-traced MP afferents within L3 and L4 DRG during knee compression in sham and cancer animals. Pressure was increased in 50-mm Hg steps every 10 seconds (see methods for more). Data represent the mean ± SEM of n = 14 cells from 3 animals (sham) and 43 cells from 5 animals (cancer). Repeated-measures ANOVA: NS, nonsignificant (E). Fluorescence intensities of all responding TD+ neuronal cell bodies to knee compression. Pressure was increased in 50-mm Hg increments every 10 seconds. Data represent the mean ± SEM of n = 9 cells (cancer) from 3 animals. No between-group comparison was performed because in sham, no cells responded (F). ANOVA, analysis of variance; CIBP, cancer-induced bone pain; DRG, dorsal root ganglia.

Approximately 13% of traced tibial cavity afferents responded to knee compression with no differences observed between sham-operated and CIBP rats (Fig. 7A) suggesting that newly recruited afferents in CIBP may originate from outside of the bone (Fig. 7B). High-pressure stimulation (around 200 mm Hg) was required to engage FB responders and GCaMP6s fluorescence intensity from bone afferents after the knee compression was equally unaffected by the presence of cancer (Fig. 7C).

To investigate whether periosteum and muscle afferents (rather than bone afferents) are sensitised in CIBP rats, muscle and periosteum (MP) afferents were traced using injection of adeno-associated virus–retrograde virus expressing tdTomato outside the tibia (Fig. 7D). Virtually, no MP traced cells from the sham-operated rats responded to mechanical stimulation (Fig. 7E). In CIBP rats, however, 20% of traced MP afferents responded to knee compression (RM-ANOVA [vs sham]: F_1, 55_ = 2.868, *P* = 0.096). As with total DRG cell analysis, the number of responders increased with compression intensity (Fig. 7E). Fluorescence intensity of MP afferents after knee compression increased with the pressure surge in cancer animals (Fig. 7F).

## 4. Discussion

In the clinic, CIBP often manifests as a result of musculoskeletal compression, and mechanical pain is a commonly reported pain symptom.^4,7,11,17,19^ Given the paucity of analgesic relief from currently available therapies, it is vital to identify new therapeutic avenues of pain alleviation for patients with CIBP.

In this study, using a rat model of CIBP, behaviour was related to tumour progression for up to 14 days after surgery. Corresponding to trabecular bone destruction, CIBP rats showed behavioural hypersensitivity from day 7 onwards. By day 14, cancer progression produces some erosion of the cortical surface of the affected bone, but no complete fracture of the bone occurred at any point studied. Nonetheless, some cancer-derived products, including tumour cells themselves, are likely to leak outside the bone. However, *post mortem*, we did not observe macroscopic tumour growth at any location outside a priori injected tibiae. This is in line with other studies using similar rodent models.^1,14,20,35^ Because mechanical hypersensitivity and cortical bone destruction were evident most significantly in the late CIBP stage, bone afferent physiology was subsequently analysed in this experimental group (and equivalent sham-operated animals) only using in vivo imaging of genetically encoded calcium indicator GCaMP6s. This technique provided an opportunity to image large populations of sensory primary afferents.

We observed a three-fold increase in the number of pressure responders in cancer conditions, and these DRG cells were analysed in more detail. How is modality-reflecting information encoded by sensory neurons? To stratify whether the averaged DRG neuronal responses to mechanical stimulation reflected uniform or more complex afferent firing pattern characteristics, we used Markov Cluster Analysis (MCA), an algorithm that is widely used in genetics to cluster sizeable expression data sets as a function of biological relevance. Our results led us to propose that the most abundant “ramp” cluster likely reflects graded frequency coding of compressive forces (similarly to heat^37^), which is maintained in both health and disease.^28^ There were significantly more cells in the mid, and less in the low, clusters in CIBP rats compared with sham-operated animals suggestive of the recruitment of nociceptive, mechanically responding cells. A decrease in low cluster cells could be suggestive of an inhibition of Aβ fibres, corresponding to the numbness experienced by some patients, or the loss of this fibre type in CIBP rats. The lack of difference in the number of responders in the movement clusters suggests that no one limb position is particularly painful, simply that movement alone engages more cells in CIBP rather than sham-operated rats.

Interestingly, in CIBP rats, the increased number of cells that responded to knee compression were small to medium-size, in contrast to results obtained from sham animals where pressure activated mainly medium-size neurons. The latter is in keeping with previous literature which suggests that in healthy animals, noxious compression is encoded preferentially by myelinated Aδ nociceptors.^23^ The result from the CIBP rats was suggestive of small nociceptive afferent recruitment. Previously, “silent” nociceptive afferents were proposed to innervate deep body structures,^12,27,30^ and recently, a potential mechanism for the “unsilencing” of silent nociceptors by NGF-TrkA-Piezo2 was proposed.^27^ Considering our observed increase in the number of responders in cancer conditions, as well as the fact that the CIBP is NGF-dependent,^1,34^ it is reasonable to predict that silent nociceptors could significantly contribute to the development of pain in the CIBP rats. This theory is supported by our observation that the increase in sensory afferent response (by means of the robust recruitment of new cells, likely belonging to the “Mid” cluster) was not due to increased individual afferent activity, but rather due to the number of afferents activated. Indeed, recruitment of previously “silent” nociceptive afferents has been reported previously in inflammatory conditions.^13^ Silent nociceptors describe a type of sensory afferents that fire action potentials after electrical stimulation of the receptive field but are otherwise insensitive to noxious mechanical stimuli. This type of afferent is particularly abundant in deep body structures and extremely rare in rodent skin.^38^ Thus, for example, the colon, knee joint, urinary bladder, or muscles were shown to be richly innervated by silent nociceptors, with current estimations suggesting that, in mice, up to 50% of all deep afferents are “silent”.^12,27,30^ These afferents can be sensitised by variety of inflammatory mediators and as a result gain the ability to respond to mechanical stimuli.^12,13,27,30^

Bone afferents were traced with Fast Blue (FB) to deduce the origin of increased afferent activity. We also investigated whether the neurons innervating the tibial cavity expressed piezo-type mechanosensitive ion channel component 2 (Piezo2) and TrkA. As observed previously,^23,24,27^ both proteins were present on the bone afferents (unpublished observations). To extend this finding, we investigated the functional responses of FB-traced intratibial afferents. Previously, these fibres were shown to respond to intraosseous pressure in healthy animals,^23,24^ while NGF has been shown to sensitize mechanically activated bone nociceptors.^24^ In this study, sensory cells innervating the tibial cavity were revealed to respond to whole-limb mechanical stimulation, as demonstrated previously by electrophysiology.^23,24^ However, no additional recruitment was detected in this neuronal population suggesting that the additional nociceptors in the late cancer stage originated from outside of the bone.

For pressure stimulation and examination of proprioceptive responses by gently moving the limb along the body axis in 5 consecutive flexion–extension cycles, results were analysed using in-house R scripts. Very stringent criteria were applied to select responses: fluorescence intensity was counted as a positive response when an average signal reached 70% above baseline fluorescence plus 4 SDs as documented previously.^3^ A striking difference in the number of neurons recruited between sham and CIBP groups, especially after the knee compression (which mostly covered the tumour-growth area), was clear. Electrical activation of the receptive field was not tested in this study. Because silent nociceptors are classically defined on the basis of their responsiveness to electrical but not to mechanical stimuli (unless “unsilenced”), we relied on the direct comparison of the number of responding afferents to the chosen receptive field stimulation with the pressure ramp and their small sizes.

The major methodological limitation of the imaging sensitivity is a low sampling rate (typically 2-4 Hz) together with the lack of electrical activation of silent nociceptors in the control group (sham-operated animals). Therefore, whether the information is encoded in different temporal forms of impulses needs to be verified using methodologies offering higher temporal resolution (ie, electrophysiology). However, considering relatively slow kinetics of GCaMP6s (as compared to the length of typical action potential), the building-up intracellular calcium concentration with each action potential fired will likely reflect summation of impulses fired in response to the pressure ramp. Therefore, the increase in fluorescence with the continuous increasing stimulus would likely approximate the increase in firing frequency.

Because bone afferents themselves did not seem to be the neurons responsible for sensitization, a viral approach was used to label MP afferents. They were shown to be silent in sham-operated animals, but responsive in cancer conditions. This suggests that in the late stage of the disease, cancer induces employment of nociceptors from bone surroundings, rather than the bone cavity itself. The anatomical results must be interpreted with care. Virally delivered tdTomato expression levels around the bone remained low, and the amount of virus used was limited to ensure specificity and to avoid off-target labelling in contralateral DRG. This meant that the cell numbers were low compared with the rest of the analyses, although still considerably higher than those that can be achieved in electrophysiological studies from the same number of animals.

## 5. Conclusion

The increase in the number of mechanically responding cells, rather than the increase in individual cell sensitivity, likely translates to the mechanical hypersensitivity observed in both CIBP rats and patients with CIBP. Primary afferents respond differently according to the mechanical stimulus, suggestive of specific differential coding of pressure and proprioception (graded vs combinatorial) by the somatosensory neurons. In sham animals, pressure is likely encoded by means of the frequency change, whereas in advanced bone cancer conditions, frequency coding is largely dominated by population coding, which may itself result from the robust recruitment of silent nociceptors.

## Conflict of interest statement

The authors have no conflicts of interest to declare.

## Appendix A. Supplemental digital content

Supplemental digital content associated with this article can be found online at http://links.lww.com/PAIN/A994 and http://links.lww.com/PAIN/A993.
